# Patterns and Outcomes in the Management of Uterine Fibroids: A Hospital-Based Retrospective Study

**DOI:** 10.7759/cureus.81984

**Published:** 2025-04-10

**Authors:** Umm E Habiba, Netasha Nazar, Kashaf Fatima, Asad Ur Rehman, Aneeqa Ashraf, Mamoona Kouser, Noor Alfardan, Yusuf Yusuf, Maryyam Islam

**Affiliations:** 1 Obstetrics and Gynecology, District Headquarters (DHQ) Hospital Okara, Okara, PAK; 2 Obstetrics and Gynecology, Lady Willingdon Hospital, Lahore, PAK; 3 Obstetrics and Gynecology, Bahawal Victoria Hospital, Bahawalpur, PAK; 4 Neurosurgery, Combined Military Hospital Lahore, Lahore, PAK; 5 Obstetrics and Gynecology, Wythenshawe Hospital, Wythenshawe, GBR; 6 Obstetrics and Gynecology, Pakistan Air Force (PAF) Hospital Islamabad, Islamabad, PAK; 7 General Medicine, King Hussein Medical Center, Amman, JOR; 8 Surgery, Harrogate District Hospital, Harrogate, GBR; 9 Research and Innovation, Shalamar Medical and Dental College, Lahore, PAK

**Keywords:** clinical symptoms, medical managment, patients, reproductive, uterine fibroids

## Abstract

Background: Uterine fibroids are common benign tumors that can cause significant symptoms such as heavy bleeding, pelvic pain, and reproductive issues.

Objective: The aim of this study was to evaluate the clinical patterns, treatment modalities, and patient outcomes associated with uterine fibroids.

Methods: This retrospective study was conducted at DHQ Hospital Okara, Okara, Pakistan, from June 2024 to December 2024. Data were collected from the medical records of 355 patients diagnosed with symptomatic uterine fibroids. Women who had confirmed uterine fibroids diagnosed through ultrasound were included in the study. Women who received treatment for uterine fibroids and patients with incomplete medical records were excluded from the study. Data were collected from medical records. The demographic information included age, marital status, parity, and reproductive history.

Results: The majority of patients were aged between 35 and 50 years (69.9%), with a mean age of 41.2 ± 7.83 years. Most patients were premenopausal (231, 65%) and multiparous (266, 74.9%). The most common symptoms were menorrhagia (248, 69.9%) and pelvic pain (195, 54.9%). The most frequent treatment modality was hormonal therapy (142, 40%), followed by surgical management (89, 25.1%) and minimally invasive procedures (53, 14.9%). Symptom improvement was highest in the minimally invasive group (92, 80%), while surgical treatments showed high patient satisfaction (64, 90%).

Conclusions: It is concluded that the treatment of uterine fibroids is largely influenced by factors such as age, fibroid size, and the severity of symptoms.

## Introduction

Uterine fibroids are classified based on their location within the uterus, which significantly influences the symptoms experienced by the patient and the approach to treatment. Uterine fibroids are indeed very common, with studies suggesting that over 70% of women have at least one fibroid when examined through autopsy series. The four main types of fibroids include those categorized as intramural, subserosal, and submucosal, as well as those classified as pedunculated fibroids [1]. Medical conditions arising from intramural fibroids inside the uterine muscle wall occur regularly and result in menstrual irregularities as well as pelvic pressure symptoms. The outer part of the uterus contains subserosal fibroids, which are often asymptomatic but can lead to discomfort and changes in the surrounding organ structures. The rarest type of fibroid is submucosal since they rise beneath the endometrial lining, yet this condition triggers heavy menstrual bleeding combined with reproductive complications and miscarriage events [2]. Pedunculated fibroids attached to the uterus by a stalk exist in two forms, depending on their original location as either subserosal or submucosal [3].

The location or size of fibroids may determine whether symptoms develop, since smaller ones, particularly those in non-spatial locations, cause no symptoms. People who suffer from fibroids might experience symptoms that diminish their lifestyle quality. Heavy menstrual bleeding, known as menorrhagia, stands as one of the main symptoms that affect many women since it causes extensive periods and related fatigue and anemia [4]. Larger or multiple fibroids tend to cause frequent pelvic pain and a sensation of pressure in affected patients. Such symptoms create daily disruptions and result in severe discomfort for the patient. Some fibroids create pressure on the bladder, rectum, and spinal nerves to produce urinary frequency, constipation, and back pain. Uterine fibroids often cause infertility and pregnancy complications that result from cavity deformation and placental attachment, as well as abnormal embryo placement [5]. The appearance of fibroids increases the risk for both miscarriage and preterm labor, especially when the fibroids occupy submucosal positions. The presence of small fibroids among women does not necessarily cause fertility problems because many women with this condition still get pregnant [6].

A physician begins diagnosing uterine fibroids by performing a complete physical checkup with detailed history taking, followed by using imaging tests. The most widely used diagnostic tool for fibroid examination is pelvic ultrasound because it offers detailed visualization as well as cost-effective and non-invasive features. Hospital staff use MRI imaging to assess fibroids with precision during treatment selection processes [7]. Healthcare providers can use hysteroscopy to directly visualize submucosal fibroids and examine the uterine cavity to assess whether these tumors are contributing to infertility or recurrent pregnancy loss. The treatment strategies for uterine fibroids depend on multiple health factors, including the fibroids' dimensions, their positions, their total number, the patient's symptom strength, and her age and pregnancy ambitions. Monitoring for changes in fibroids represents a suitable approach for women who have mild symptoms [8]. Various treatment choices are available to women who face serious fibroid symptoms [9]. Medical management strives primarily to handle the symptoms, especially heavy bleeding, along with pain. The medical treatment of menstrual bleeding includes oral contraceptive drugs together with progestins and intrauterine devices (IUDs), which control cycle periods and minimize bleeding. Doctors may use gonadotropin-releasing hormone (GnRH) agonists to reduce the size of fibroids because they create a short-term menopausal condition that lowers both estrogen and progesterone levels [10].

The objective is to evaluate the clinical patterns, treatment modalities, and patient outcomes associated with uterine fibroids.

## Materials and methods

This retrospective study was conducted at DHQ Hospital Okara, Okara, Pakistan, from June 2024 to December 2024. Data were collected from the medical records of 355 patients diagnosed with symptomatic uterine fibroids. Women who had confirmed uterine fibroids diagnosed through ultrasound were included in the study. Women who received treatment for uterine fibroids and patients with incomplete medical records were excluded from the study.

Data collection

Data were collected from medical records. The demographic information consisted of patient age, alongside marital status, reproductive history, and parity. The physicians also documented symptoms shown by patients, including menstrual abnormalities as well as pelvic pain and urinary complaints and infertility problems. Healthcare providers used pelvic ultrasound together with MRI and hysteroscopy to confirm diagnosis and evaluate fibroid dimensions and types and locations for each patient. Additionally, the data included patient outcome indicators such as symptom improvement, post-treatment complications, and overall treatment results. The assessment of patient treatment satisfaction, symptom severity changes, and life quality modifications incorporated systematically designed questionnaires that measured these factors before and after therapy.

Data analysis

Data were analyzed using IBM SPSS Statistics for Windows, Version 26 (Released 2019; IBM Corp., Armonk, New York, United States). Descriptive statistics were used to summarize the demographic characteristics of the study population, clinical presentations, and treatment modalities. Frequency distributions were calculated to identify the most common symptoms and types of fibroids diagnosed. To evaluate the differences between treatment groups (medical, surgical, or minimally invasive), t-tests were conducted. A p-value of less than 0.05 was considered statistically significant.

## Results

Data were collected from 355 patients with a mean age of 41.2 ± 7.83 years, with the majority (248, 69.9%) falling in the 35-50 years age range. Most patients were premenopausal (231, 65%) and multiparous (266, 74.9%), with a significant proportion being married (284, 80%). The most common clinical symptoms included menorrhagia (heavy bleeding) in 248 (69.9%) patients, followed by pelvic pain in 195 (54.9%) and urinary symptoms in 89 (25.1%). A smaller percentage experienced infertility or other reproductive issues (53, 14.9%) and gastrointestinal symptoms (5.1%) (Table 1).

**Table 1:** Demographic characteristics of study population (n = 355) Data are represented as frequency (n) and percentage (%) for categorical variables and mean ± standard deviation (SD) for continuous variables. A p-value of <0.05 was considered statistically significant.

Characteristic	Frequency (n)	Percentage
Age range		
18-34 years	50	14.1
35-50 years	248	69.9
51-58 years	57	16.0
Mean age	41.2 ± 7.83 years	
Reproductive status		
Premenopausal	231	65.0
Postmenopausal	124	35.0
Parity		
Nulliparous	89	25.1
Multiparous	266	74.9
Marital status		
Married	284	80.0
Unmarried	71	20.0
Clinical symptom		
Menorrhagia (heavy bleeding)	248	69.9
Pelvic pain	195	54.9
Urinary symptoms (frequent urination)	89	25.1
Infertility/reproductive issues	53	14.9
Other symptoms (constipation, GI issues)	18	5.1

The most common treatment was hormonal therapy (e.g., oral contraceptives, IUD), used by 142 patients (40%), followed by GnRH agonists in 35 patients (10%). Among the surgical management options, myomectomy was the most frequent procedure, performed in 89 patients (25.1%), while hysterectomy was performed in 71 patients (20%). Minimally invasive options included uterine artery embolization (UAE), used by 53 patients (14.9%), and focused ultrasound, performed in 18 patients (5.1%) (Table 2).

**Table 2:** Treatment modalities (n = 355) The data are represented as frequency (n) and percentage (%) for categorical variables. GnRH: gonadotropin-releasing hormone; IUD: intrauterine device

Treatment modality	Frequency (n)	Percentage
Medical management		
Hormonal treatment (e.g., oral contraceptives, IUD)	142	40.0
GnRH agonists	35	10.0
Surgical management		
Myomectomy	89	25.1
Hysterectomy	71	20.0
Minimally invasive procedures		
Uterine artery embolization (UAE)	53	14.9
Focused ultrasound	18	5.1

Sixty-seven (75.3%) patients undergoing surgical treatment (myomectomy/hysterectomy) reported complete or near-complete resolution, while 85 (60%) of those receiving medical treatment and 92 (80%) of those receiving minimally invasive procedures (UAE, focused ultrasound) saw improvement. For patient satisfaction, 64 (90%) patients who underwent surgery were highly satisfied, 70 (80%) patients who had minimally invasive procedures were highly satisfied, and 60% of those receiving medical treatment expressed satisfaction (Table 3).

**Table 3:** Outcomes and patient satisfaction The data are represented as frequency (n) and percentage (%) for categorical variables. UAE: uterine artery embolization

Outcome/patient satisfaction	Frequency (n)	Percentage
Symptom improvement		
Complete or near-complete resolution (surgical)	67	75.3
Symptom improvement (medical)	85	60.0
Symptom improvement (minimally invasive)	92	80.0
Patient satisfaction		
Highly satisfied (surgical)	64	90.0
Highly satisfied (minimally invasive)	70	80.0
Satisfied (medical)	85	60.0
Recurrence of fibroids (surgical)		
Myomectomy recurrence (within 1 year)	9	10.1
Complications		
Surgical complications (myomectomy/hysterectomy)	12	3.4
Minimally invasive (UAE, focused ultrasound)	7	5.2

The age distribution across treatment groups showed no significant differences, except for the 51-58 years age group, where surgical management was more common (p = 0.04). In terms of fibroid size, significant differences were observed. Medical management was more common for smaller fibroids (<4 cm), while surgical management was more common for fibroids between 4 and 6 cm. No significant differences were found for larger fibroids (>6 cm) (Table 4).

**Table 4:** Comparison of age group and fibroid size distribution by treatment modality The data are represented as frequency (n) and percentage (%). The statistical analysis performed for comparing categorical variables across groups is the chi-square test. A p-value of <0.05 was considered statistically significant.

Characteristic/group	Medical management (n = 142)	Surgical management (n = 160)	Minimally invasive (n = 53)	Total (n = 355)	p-value	χ² value
Age group						
18-34 years	24 (16.9%)	20 (12.5%)	6 (11.3%)	50 (14.1%)	0.32	2.21
35-50 years	96 (67.6%)	106 (66.3%)	46 (86.8%)	248 (69.9%)	0.15	4.92
51-58 years	22 (15.5%)	34 (21.2%)	1 (1.9%)	57 (16.0%)	0.04	10.23
Fibroid size (cm)						
<4 cm	70 (49.3%)	45 (28.1%)	19 (35.8%)	134 (37.8%)	0.01	11.89
4-6 cm	45 (31.7%)	75 (46.9%)	25 (47.2%)	145 (40.9%)	0.02	9.92
>6 cm	27 (19.0%)	40 (25.0%)	9 (17.0%)	76 (21.4%)	0.13	3.50

## Discussion

The results of the study reveal key insights into the treatment patterns and patient characteristics in those presenting with uterine fibroids. The analysis of age differences revealed that treatment frequencies were similar across most age groups, with the exception of the 51-58 years demographic, where surgical treatments were more commonly applied. The selection of medical treatments showed different preferences according to fibroid dimensions [11]. Small fibroids measuring less than 4 cm received primary medical treatment since hormone therapies proved effective for managing mild symptoms in this category. Medical interventions alone were less prevalent for treating fibroids in the 4-6 cm size group since doctors often selected surgical methods to address such moderate to severe symptom-causing fibroids. Larger fibroids exceeding 6 cm showed a similar distribution of treatment approaches, as they generally need individualized management plans for patients' clinical situations, alongside their reproductive plans and general health status [12]. The findings demonstrate that treatment selection depends on fibroid size and patient age because smaller tumors are suitable for medical interventions, whereas larger conditions need surgical removal. Patients older in age tend to choose surgery or minimally invasive surgery when their fibroid symptoms intensify. The study demonstrates why specific treatment options should depend on each patient's medical characteristics in addition to the observations about patient age and fibroid dimensions [13].

Research suggests medical treatment receives higher priority from patients who have smaller fibroids because these fibroids generally cause minimal symptoms. Hormonal therapies that include oral contraceptives together with IUDs successfully manage symptoms of menorrhagia and pelvic pain, thus becoming preferred choices for these patients. Non-surgical interventions remain the standard to treat less symptomatic cases due to their lower risk level. A study conducted by Lethaby et al. (2000) explained that the treatment group consisted of women with both uterine fibroids and heavy menstrual bleeding, while patients only suffering from uterine fibroids remained untreated. Pain emerged as the main documented symptom alongside heavy menstrual bleeding among women with uterine fibroid disorder [14]. According to data, many physicians choose surgical treatment for fibroids within a size range of 4-6 cm since such fibroids produce compelling symptoms that require extensive medical intervention; thus, surgical procedures become the preferred course of action.

The surgical intervention of myomectomy finds preference when treating patients who desire preservation of their fertility status, together with symptom relief, particularly when they are either young or intend to retain childbearing potential [15]. Hysterectomy continues as a favored surgical option when treating older women and patients who experience enduring fibroid-related symptoms due to the higher incidence of postmenopausal patients receiving surgical treatments. Shaikh et al. (2020) conducted an observational study and concluded that menorrhagia served as the most common clinical presentation among the patients who received a hysterectomy surgery as their primary treatment, despite being the only permanent solution for uterine fibroids [16]. A study conducted by Bano et al. (2021) also explained that fibroid uterus represents the most prevalent benign tumor of female reproductive organs that affects women of childbearing age, fertility outcomes, and the general health of females. This research confirms how a major number of patients need surgical intervention. Patients need surgical procedures to address their conditions since a fibroid uterus presents through multiple medical manifestations, while medical treatments frequently prove ineffective [17].

Limitations

There are some limitations of our study. The use of retrospective data collection methods in this research presents limitations because it introduces selection and data bias into the investigation. This observational research did not measure vital elements that might control study results because it failed to consider fibroid symptom length or comorbidities, or previous therapeutic interventions. The results might lose accuracy due to the method of estimating both patient age and fibroid dimensions based on midpoint calculations and provided ranges. Long-term follow-up information is missing, which prevents us from determining how well the treatment results persist along with recurrence risks throughout the period.

## Conclusions

It is concluded that the treatment of uterine fibroids is largely influenced by factors such as age, fibroid size, and the severity of symptoms. Medical management is most effective for smaller fibroids with milder symptoms, while surgical and minimally invasive procedures are more commonly employed for larger fibroids or more severe cases. The study highlights the importance of individualized treatment plans, with older patients and those with larger fibroids tending to opt for surgical interventions.
